# First-principles study of highly sensitive graphene/hexagonal boron nitride heterostructures for application in toxic gas-sensing devices

**DOI:** 10.1039/d3ra08017j

**Published:** 2024-02-06

**Authors:** Viet Bac T. Phung, Ba Lich Pham, Nguyen Vo Anh Duy, Minh Triet Dang, Thi Nhan Tran, Quang-Huy Tran, Thi Theu Luong, Van An Dinh

**Affiliations:** a Center for Environmental Intelligence and College of Engineering & Computer Science, Vin University Hanoi 100000 Vietnam bac.ptv@vinuni.edu.vn; b Institut de Chimie Physique, Faculté des Sciences d’Orsay, Université Paris-Saclay Orsay 91405 France; c FPT University Can Tho Campus, 600 Nguyen Van Cu Street, Ninh Kieu Can Tho Vietnam; d School of Education, Can Tho University 3-2 Road Can Tho Vietnam; e Faculty of Fundamental Sciences, Hanoi University of Industry 298 Cau Dien Street, Bac Tu Liem District Hanoi 100000 Vietnam; f Faculty of Physics, Hanoi Pedagogical University 2 Phuc Yen Vinh Phuc Vietnam; g Hoa Binh University Bui Xuan Phai Str., My Dinh II, Nam Tu Liem Hanoi 100000 Vietnam; h Department of Precision Engineering, Graduate School of Engineering, Osaka University 2-1 Yamadaoka Suita Osaka 565-0871 Japan divan@prec.eng.osaka-u.ac.jp

## Abstract

Graphene-based sensors exhibit high sensitivity, fast response, and good selectivity towards toxic gases but have low mechanical stability. The combination of graphene and two-dimensional hexagonal boron nitride (h-BN) is expected to increase the mechanical stability and enhance the adsorption performance of these gas sensors. Using first-principles calculations, we demonstrate that two-dimensional graphene/h-BN double layers can be used as good substrates for gas sensors with a small lattice mismatch of only 1.78%. Moreover, the presence of a h-BN layer widens the band gap by about 38 meV and considerably increases the work function, thus positively affecting the gas adsorption performance. Although these graphene/h-BN heterostructures do not change the physical adsorption mechanism of these sensors concerning the graphene-based materials, these bilayers significantly enhance the sensitivity of these sensors for detecting CO_2_, CO, NO, and NO_2_ toxic gases. Particularly, compared to the pristine graphene-based materials, the gas adsorption energies of graphene/h-BN increased by up to 13.78% for the adsorption of NO, and the shortest distances between the graphene/h-BN substrates and adsorbed gas molecules decreased. We also show that the graphene/h-BN heterostructure is more selective towards NO_*x*_ gases while more inert towards CO_*x*_ gases, based on the different amounts of charge transferred from the substrate to the adsorbed gas molecules. Using the non-equilibrium Green functions in the context of density functional theory, we quantitatively associated these charge transfers with the reduction of the current passing through these scattering regions. These results demonstrate that graphene/h-BN heterostructures can be exploited as highly sensitive and selective room-temperature gas sensors for detecting toxic gases.

## Abstract 2

Density Functional Theory (DFT) was employed to investigate the sensitivity and selectivity of graphene/h-BN heterostructures for application in toxic gas sensing devices.

## Graphical abstract

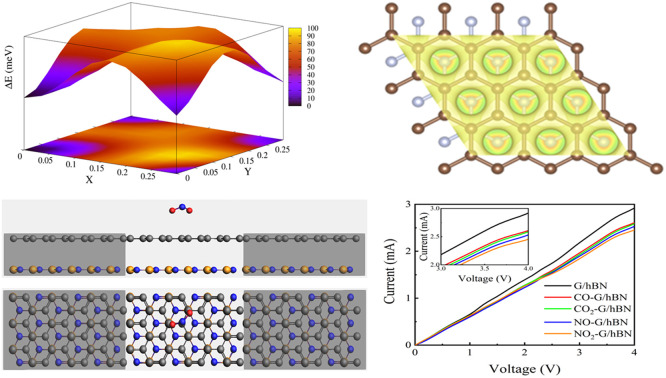

## 1. Introduction

Highly sensitive and selective toxic gas sensors have received great attention for their industrial and pharmaceutical applications. Typically, most conventional low-cost metal oxide gas sensors work at high temperatures,^1^ raising significant concerns in terms of energy consumption for operation and safety issues. Embedding noble metals (Pd, Pt, Au, Ag, and so on) on the surface of metal oxides could reduce the operating temperature of gas sensors because these noble metal atoms act as catalysts promoting dissociation and reducing the activation energy of the adsorbed gas molecules.^2,3^ Two-dimensional (2D) materials such as graphene, monolayer phosphorene, and transition metal dichalcogenide compounds are considered good candidates for high-performance room temperature gas sensors, which can be substituted for traditional metal oxide materials.^4–8^

Graphene, a single layer of graphite formed by hexagonal structures, has been of great interest for both academic and industrial applications since it was first discovered in 2004 by the group of A. Geim and K. Novoselov.^9^ Its low-energetic electronic structure, resembling two-dimensional dirac massless fermion dynamics at the speed of light *c*, replaced by the Fermi velocity *v*_f_ ≈ *c*/300, makes ultrarelativistic physics observable for this material.^9,10^ Graphene has been considered a promising material for flexible and transparent electronic applications owing to its extraordinary electronic and mechanical properties resulting from a perfect hexagonal crystal lattice. However, it is challenging to make graphene into nanoelectronic devices due to the mechanical instability of a freestanding graphene layer. Recent attempts^11–16^ to assemble graphene layers on the top of silicon dioxide (SiO_2_), gallium arsenide (GaAs), indium gallium arsenide (InGaAs), and hexagonal boron nitride (h-BN) layers have been widely studied to significantly surmount the mechanical instability of pristine graphene and to enhance the electronic and optical properties of graphene.^9^

In the field of gas sensing, research has been devoted to expanding these 2D materials into van der Waals (vdW) 2D heterostructures to directly control the electronic structure of graphene-like materials, such as actively opening up the graphene band gaps.^12,14,17^ However, to successfully assemble a graphene layer on top of a substrate, the chosen substrate should share some geometrical similarity to graphene to maintain its mechanical and electronic stability. Hexagonal boron nitride is an excellent candidate for this purpose because graphene and h-BN monolayers share a similar lattice structure with a lattice mismatch of only ∼1.5%, a smooth surface without any significant charge accumulators, and high-temperature stability.^13,14,18–21^

Despite many efforts to explore the electronic structure of graphene on an h-BN substrate, the influence of h-BN layers on the adsorption behavior of graphene towards adsorbed toxic gas molecules is still poorly understood. Herein, we have used first-principles methods based on density functional theory (DFT)^22,23^ to investigate the structural stability, and electronic and electron transport properties of graphene/h-BN heterostructures upon the adsorption of toxic gas molecules. First, we performed DFT optimization calculations of each single layer and then calibrated the energy profile of these heterostructures as a function of the interlayer distance between these two layers to obtain the most relaxed geometric configuration of graphene/h-BN heterostructure. The electronic properties of the most stable structure were compared and discussed with those of the single layers. We then investigated the adsorption mechanism of pristine graphene and graphene/h-BN upon the adsorption of toxic gases, including CO_2_, CO, NO, and NO_2_. To increase the reliability of these electronic calculations, we took into account the vdW interactions between these substrates and the adsorbed molecules. To evaluate the selectivity of graphene/h-BN heterostructures as advanced toxic gas sensors, we also employed non-equilibrium Green's function formalism with density functional methods to calculate the electron transport properties across these adsorbed systems.

## 2. Computational method

In this work, all periodic DFT calculations were performed using the projector-augmented wave (PAW) method^24^ implemented in the Vienna *Ab initio* simulation package (VASP).^25,26^ To resolve the interlayer interactions between graphene and h-BN, we included the van der Waals dispersion corrections and evaluated multiple exchange–correlation functionals in the non-local vdW functional family, such as optPBE-vdW,^27^ revPBE-vdW,^28^ and vdW-DF2.^29^ Such vdW corrections provide reasonable comparisons to experimental observations for heterostructures. The integration in the Brillouin zone was employed using the Gamma-centered 6 × 6 × 1 and 12 × 12 × 1 *k*-points for the geometry optimization and electronic calculations, respectively, with an energy cutoff of 520 eV. Lattice parameters and atom coordinates were optimized until the Hellmann–Feynman force acting on each atom was less than 0.01 eV Å^−1^ and the energy difference between two consecutive steps was less than 10^−5^ eV. To avoid the atomic interactions between the imaged periodic systems, a vacuum of 22 Å thickness was employed between the neighboring layers. All visualizations of atomic configurations and charge density presented in this work have been visually displayed by the VESTA package developed by K. Momma and F. Izumi.^30^

To evaluate the adsorption mechanism of the toxic gas molecules on these heterostructures, a 3 × 3 supercell of graphene/h-BN heterostructures was adopted. Upon adsorption, the precise molecule positions and orientations are crucial for evaluating the optimal geometrical configurations. Thus, we employed the high-resolution computational DFT-based Nanoscope tool^31–33^ to scan through all possible configurations to obtain the potential energy surface (PES) and the binding energy profile. This tool uses the adsorbate as a scanning tip, moves the gas molecule across the graphene or graphene/h-BN heterostructures, and rotates around its center of mass^34^ to obtain the high-resolution energy landscape. The potential energy landscape or the adsorption energy of the system can be determined as follows:^31,32^

1 *E*_ads_ = *E*_complex_ − *E*_saturation_,

where *E*_complex_ and *E*_saturation_ are the total energy of the gas + graphene or gas + graphene/h-BN heterostructures, and the total energy of the system in which the gas molecule and the graphene (graphene/h-BN) are isolated, respectively.

The binding energy of graphene/h-BN heterostructures is calculated as follows:

2 *E*_binding_ = *E*_G/h-BN_ − (*E*_G_ + *E*_h-BN_),

where *E*_G/h-BN_, *E*_G_ and *E*_h-BN_ are the energies of graphene/h-BN, pristine graphene, and isolated h-BN sheets, respectively.

To estimate the selectivity of the graphene/h-BN, we investigated the transport behaviors of graphene/h-BN upon the adsorption of toxic gases by employing non-equilibrium Green's function^35^ (NEGF) with the density functional formalisms implemented in the Amsterdam Modelling Suite^36^ to compute the transmission functions, which indicated the number of electrons transferred across the scattering region between two electrodes. The electrodes are composed of pristine graphene/h-BN bilayers with the exact size and shapes of the pristine scattering region. The transmission function *T*(*E*, *V*_b_) at an energy *E* under an applied voltage *V*_b_ is calculated as follows:^37,38^

3 *T*(*E*, *V*_b_) = Tr[*G*(*E*)*Γ*_L_(*E*)*G*^+^(*E*)*Γ*_R_(*E*)],

where *G* and *G*^+^ are the retarded and advanced Green functions of the scattering sample, respectively. *Γ*_L_ and *Γ*_R_ are the coupling functions from the left and right leads, respectively. The current *I* across the considered sample at the bias voltage *V*_b_ is obtained from the Landauer–Buttiker formula:^37,38^4
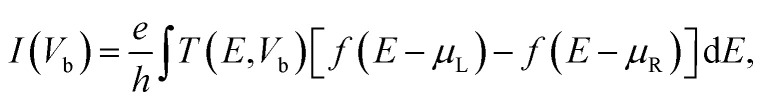
where *f*(*E* − *μ*_L_) and *f*(*E* − *μ*_R_) are the Fermi–Dirac functions of the left and right electrodes corresponding to the chemical potentials *μ*_L_ and *μ*_R_ (*μ*_L_ − *μ*_R_ = *eV*_b_), respectively; *e* is the electron charge and *h* is Plank's constant.

## 3. Results and discussion

### 3.1. The graphene/h-BN bilayer heterostructure

The lattice mismatch is a parameter that illustrates the potential to assemble a long-period moiré superstructure without rotation between two different lattices. In other words, the strain effect on the electronic properties of graphene and h-BN can be ignored if this quantity is sufficiently small. The lattice mismatch *ε*_0_ is estimated by using the following relation:5
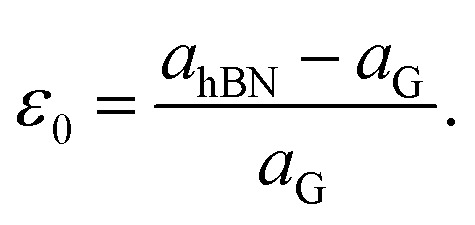


We optimized the geometric structure of graphene and h-BN sheets using the revPBE-vdW functional. The obtained lattice constants for the graphene and h-BN layers are *a* = *b* = 2.468 Å and *a* = *b* = 2.512 Å, respectively, which are in agreement with previous studies.^41,42^ Our calculated lattice mismatch between the graphene and h-BN monolayer is relatively small, *ε*_0_ ∼ 1.78% (as shown in Table 1), in excellent agreement with previous studies.^40,41,43,44^ Such a small lattice mismatch demonstrates the possibility of assembling long-range superstructures from these layers. Thus, graphene/h-BN is more beneficial for assembling superstructures as compared to the other heterostructures such as h-BN/phosphorene (5%) and silicene/graphene (<4%), and is as good as phosphorene/borophene (<2%) bilayers.

**Table 1:** The lattice mismatch between graphene and the h-BN sheet

No.	Graphene *a*_G_ (Å)	h-BN *a*_hBN_ (Å)	Lattice mismatch *ε*_0_ (%)	Reference
1	2.445	2.500	<2	39
2	2.468	2.512	2	40
3	2.468	2.512	1.78	In present work

**Fig. 1:**
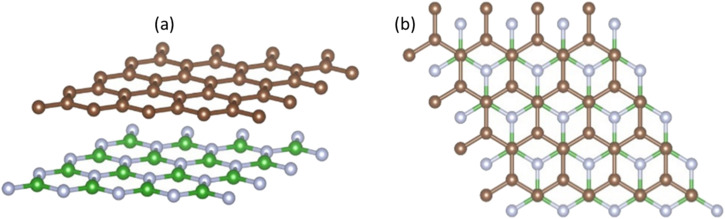
Side view (a) and top view (b) of the graphene/h-BN heterostructures with AB stacking. Boron atoms are marked in green, nitrogen in light blue, and carbon in brown.

**Fig. 2:**
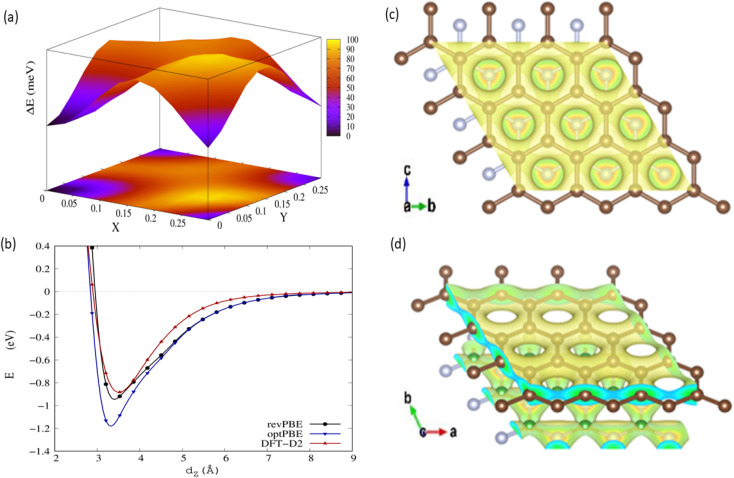
Interactions of graphene and h-BN layers. (a) Potential energy surface of graphene on h-BN. (b) The interaction energy *versus* the interlayer distance between graphene and the h-BN substrate with different vdW interaction models. Top-view (c) and side-view (d) of the charge density profile at the interface between graphene and h-BN.

**Fig. 3:**
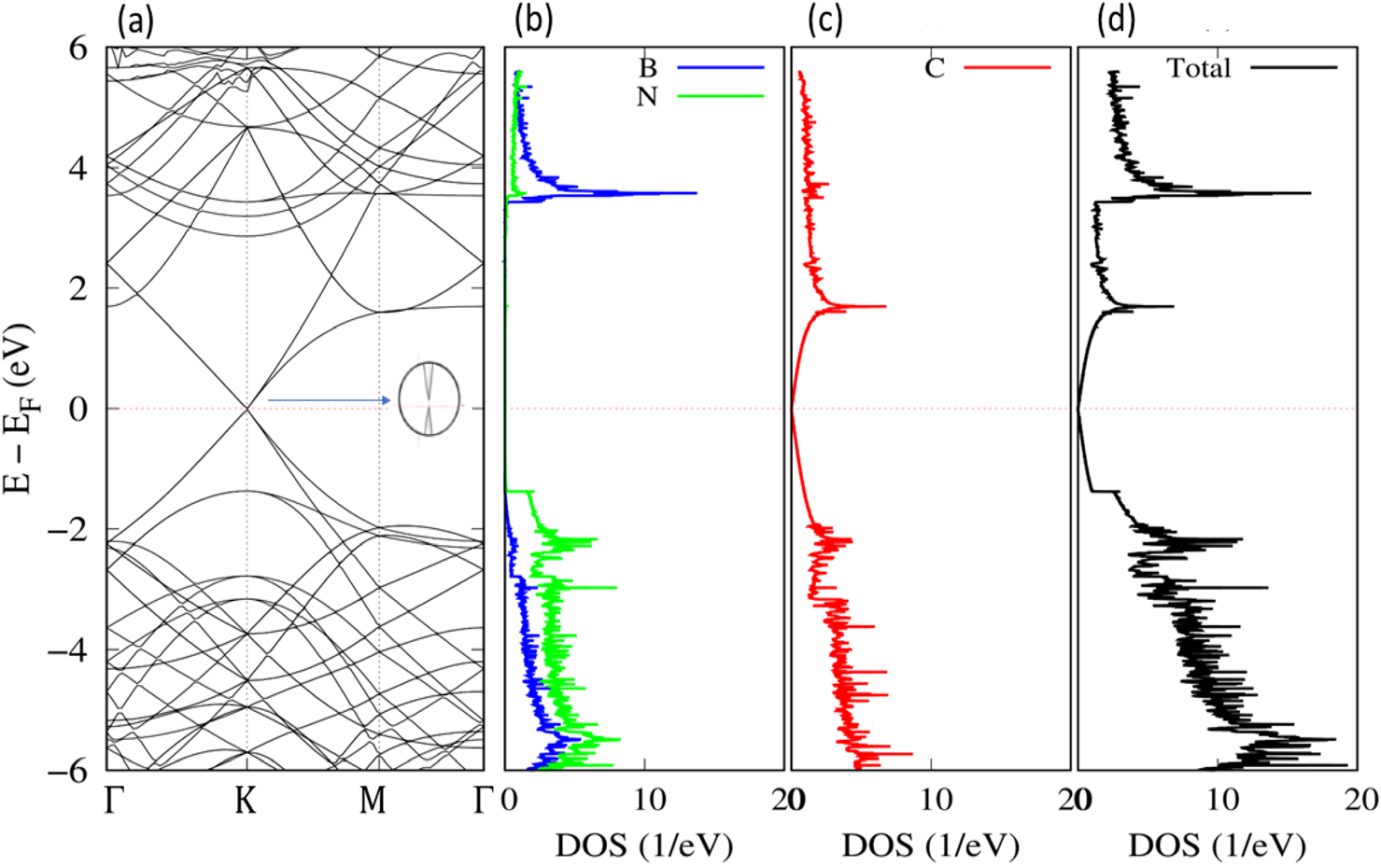
Band structure along the Γ–K–M–Γ path in reciprocal space (a) and DOS for the relaxed structure of graphene/h-BN: (b) h-BN; (c) graphene; and (d) total DOS.

We evaluated the stacking order dependence of graphene/h-BN heterostructures before adsorbing toxic gas molecules. It is known that there are three possible stacking configurations to assemble a graphene monolayer on top of an h-BN substrate: (1) AA stacking with C atoms in graphene located on top of B and N atoms; (2) AB stacking with C atoms of the upper graphene layer either on top of N atoms or at the center of h-BN hexagon structures as displayed in Fig. 1; (3) AB stacking with C atoms of the upper graphene layer either on top of B atoms or at the center of h-BN hexagons. Instead of manually placing a graphene layer on an h-BN hexagon, as mentioned above, we developed a more robust scanning method by fine-tuning the upper layer horizontally and later vertically to obtain the potential energy surface (PES). The most stable geometrical stacking configuration and the interlayer distance between the graphene and h-BN layers were determined based on the PES profile (Fig. 2(a)). The color gradient on the right of Fig. 2(a) represents the relative energy difference with respect to the energy value of the most stable configuration with *X* and *Y* being fractional coordinates based on the lattice vectors of the *XY*-plane of the graphene unit cell in the simulation model. The vertical axis Δ*E* in Fig. 2(a) displays the relative energy value. The upper color surface indicates the three-dimensional image of PES, which projected PES on the surface of a unit cell of graphene as shown by the lower one. Dark-colored regions (indicating low energy) correspond to the favorable adsorption sites of the C atom of the graphene layer on the h-BN, while brightly colored regions (exhibiting high energy) represent the less favorable sites. We found that the AB stacking with C atoms located on top of B atoms as illustrated in Fig. 1 is the most stable geometrical stacking. This approach allows the extraction of the most stable geometrical stacking configuration at high accuracy. After determining the most favorable stacking configuration, we rotated the graphene around the *z*-axis to calculate the binding energy *versus* the interlayer distance between the graphene and h-BN displayed in Fig. 2(b). Finally, we extracted the most stable configuration of a graphene layer on top of a h-BN with a minimum binding energy.

To understand the underlying mechanism of this stacking order dependence, we further examined the charge density profile of the graphene/h-BN heterostructures. As indicated in Fig. 2(c) and (d), in the graphene layer, the electron cloud mainly surrounds the C–C bonds in hexagonal honeycombs, while, in the lower h-BN layer, this cloud is mostly distributed around nitrogen atoms since these atoms have a larger electron affinity than those of boron atoms (*i.e.* electronegativity *χ*_N_ = 3.04 > *χ*_B_ = 2.04). Thus, nitrogen atoms act as anions, whereas boron atoms are cations, inducing attractive interactions towards the electron cloud above, whereas the nitrogen anions located at the center of graphene hexagons repel the graphene's electron cloud. The balance between the attractive and the repulsive forces results in the most stable AB stacking pattern.

Taking the most stable AB stacking configuration with C atoms on top of B atoms, we evaluated the equilibrium interlayer distances between graphene and h-BN and the corresponding binding energies with various exchange–correlation functionals. Table 2 presents the interlayer distances and the corresponding binding energies with multiple exchange–correlation functionals such as revPBE-vdW, optPBE-vdW, and vdW-DF2. Among these functionals, the optimized distance between h-BN and graphene obtained from the revPBE-vdW functional is in quantitative agreement with the experimental value of 3.33 Å reported in ref. 39 and 44. To evaluate the sensitivity of these bilayers for application in gas sensing devices, we further computed the work function (WF) using the revPBE-vdW functional. The calculated WF is 4.28 eV, which is quite close to the value calculated in the previous work (4.44 eV)^42^ and given by a previous experiment (4.57 eV).^45^ It was reported that the theoretical WF of h-BN is 5.986 eV,^46^ which is considerably higher than that of pristine graphene. In the presence of h-BN sheets, the WF of graphene increases considerably, from 4.281 eV to 4.507 eV, implying the modification in the graphene surface potential and interface electronic properties caused by the interaction with h-BN. The WF of graphene/h-BN is comparable to the WF values of the other common semiconductor materials such as the neutral WO_3_ monolayer (6.97 eV)^47^ and anode materials such as Cu_4_S_4_ (5.42 eV),^48^ demonstrating that these heterostructures are suitable for application in gas-sensing devices.

**Table 2:** Geometrical characteristics of graphene/h-BN heterostructures obtained from different van der Waals-DFT functionals

vdW-DFT functionals/characteristics	revPBE-vdW	optPBE-vdW	vdW-DF2
Distance *d*_*z*_ (Å)	3.252	3.250	3.552
Response length (Å)	10.052	9.313	8.921
Binding energy (eV)	0.910	1.169	0.881
Work function (eV)	4.507	4.528	4.498

### 3.2. The electronic structure of the graphene/h-BN bilayer

To investigate the influence of the h-BN substrate on the gas response of graphene, we calculated the electronic structure and density of states (DOS) of the relaxed graphene/h-BN. The electronic band structures and DOS analysis with the revPBE-vdW functional are shown in Fig. 3. The Fermi level displayed by dotted lines in Fig. 3, was set up at the highest occupied molecular orbital (HOMO) state. It is widely recognized that pristine graphene possesses a band structure with zero band gap, reducing its sensitivity and selectivity to a wide range of gases. However, the electronic band structure of graphene/h-BN exhibited a gap opening of 38 meV (see the inserted image in Fig. 3), with a binding energy of about 0.056 eV per carbon atom. The interaction with the h-BN sheet helps to open the band gap of graphene by breaking the geometric symmetry of graphene, similar to that happening when applying the strain effect,^49^ as well as doping boron or nitrogen atoms.^50^ Additionally, the separation between the HOMO and the lowest unoccupied molecular orbital (LUMO) of the h-BN layer in interaction with graphene is ∼5.2 eV, close to the HOMO–LUMO separation of pristine h-BN. The DOS image around the Fermi level of graphene/h-BN presented in Fig. 3(c) exhibits an inconsiderable change with respect to that of pristine graphene. Graphene/h-BN heterostructures retained the electronic characteristics of both graphene and h-BN.

### 3.3. The influence of the h-BN substrate on the gas adsorption of graphene

#### 3.3.1. The optimized geometric structures of the adsorption systems

**Fig. 4:**
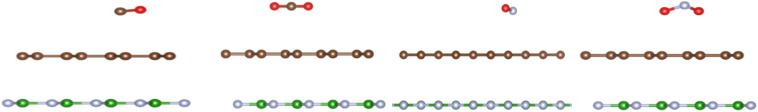
The optimized structures (from left to right) of CO, CO_2_, NO, and NO_2_ adsorbed on graphene/h-BN.

We performed calculations with the use of a computational DFT-based nanoscope to determine the most preferred geometric configurations of CO, CO_2_, NO, and NO_2_ molecules absorbed on graphene/h-BN as well as on pristine graphene. As shown in Fig. 4, the adsorbed CO_2_ molecule is likely oriented parallel to the surface of graphene/h-BN. The NO_2_ molecule adopted a configuration in which two oxygen atoms pointed downward towards the graphene surface, and atoms O, N, and O formed an inverted V-shaped pattern. The shortest distances between adsorbed gas molecules and graphene, with or without the presence of h-BN sheet, were ranked in increasing order as NO < NO_2_ < CO_2_ < CO. These distances fall within the range of 3.0–3.5 Å, surpassing the limit of chemical interaction (Table 3). This indicates the physical adsorption of the four toxic gases on graphene/h-BN as well as on pristine graphene, which also showed the adsorption of oxygen molecules in literature.^48^

**Table 3:** A comparison of adsorption parameters for toxic gas molecules on graphene/h-BN and pristine graphene using three van der Waals functionals

Gases	Characteristics	Gas on G/h-BN			Gas on the pristine graphene		
		revPBE	optPBE	DFT-DF2	revPBE	optPBE	DFT-DF2
CO	*d*(gas-G) (Å)	3.383	3.276	3.425	3.432	3.334	3.418
	*E*_ads_ (eV)	0.171	0.202	0.161	0.162	0.196	0.160
	Recovery time *τ* (ns)	0.74	2.47	0.51	0.53	1.96	0.49
	Work function (eV)	4.531	4.443	4.532	4.452	4.562	4.545
	Charge transfer (*e*)	0.007	—	—	0.008	—	—
CO_2_	*d*(gas-G) (Å)	3.361	3.320	3.535	3.373	3.312	3.536
	*E*_ads_ (eV)	0.226	0.259	0.218	0.214	0.262	0.209
	Recovery time *τ* (ns)	6.24	22.37	4.58	3.93	25.12	3.24
	Work function (eV)	4.531	4.551	4.525	4.433	4.572	4.519
	Charge transfer (*e*)	0.011	—	—	0.015	—	—
NO	*d*(gas-G) (Å)	3.117	3.095	3.312	3.119	3.106	3.234
	*E*_ads_ (eV)	0.223	0.268	0.213	0.196	0.213	0.180
	Recovery time *τ* (ns)	5.56	3.17	3.78	1.96	3.78	1.05
	Work function (eV)	4.674	4.456	4.387	4.598	4.657	4.526
	Charge transfer (*e*)	0.049	—	—	0.056	—	—
NO_2_	*d*(gas-G) (Å)	3.156	3.098	3.265	3.194	3.123	3.269
	Work function (eV)	5.648	4.352	4.498	5.668	4.364	4.535
	*E*_ads_ (eV)	0.314	0.318	0.312	0.312	0.307	0.303
	Recovery time *τ* (ns)	190	220	170	170	140	120
	Charge transfer (*e*)	0.196	—	—	0.194	—	—

#### 3.3.2. The adsorption energies and charge transfers

**Fig. 5:**
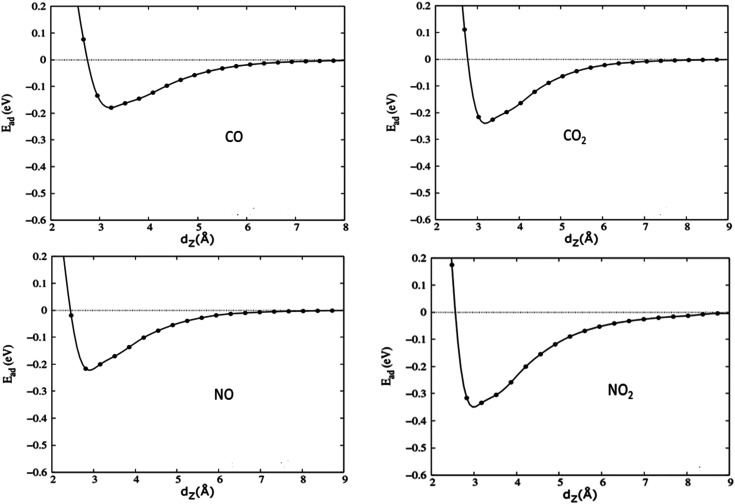
Energy profiles, which present the interaction energy *versus* the shortest distance between graphene and the adsorbed gas molecule *d*_*z*_, of four toxic gases on graphene/h-BN heterostructures calculated by the revPBE – vdW functional.

**Fig. 6:**
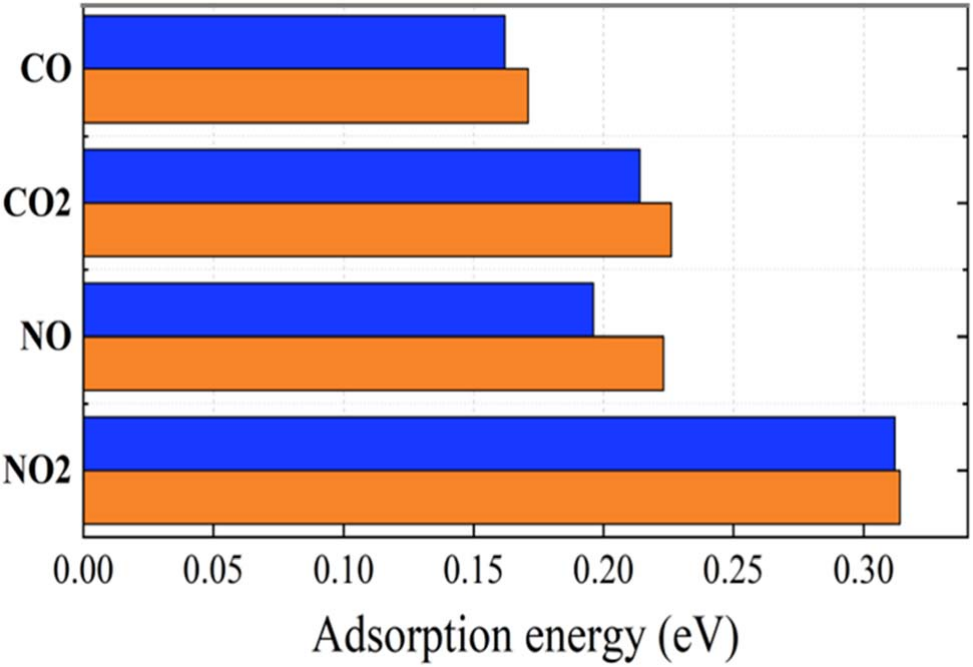
Adsorption energies of toxic gases on graphene (blue) and graphene/h-BN (red) with the revPBE -vdW functional arranged in increasing order from upper to lower.

To carefully evaluate the influence of the h-BN substrate on the gas sensitivity of graphene, we calculated the adsorption energy profiles of the gases on graphene/h-BN as well as on pristine graphene. The vdW-DFT functionals are known to accurately describe molecular adsorption. However, there are presently no benchmark calculations using highly accurate methods.^32^ Therefore, to provide the range of adsorption energies as well as probe the adsorption mechanism of toxic gases on pristine graphene and graphene/h-BN heterostructures, three vdW-DFT functionals including revPBE-vdW, optPBEvdW, and vdW-DF2 were used to calculate adsorption parameters. Table 3 shows that the adsorption energies of the gases on pristine graphene or graphene/h-BN vary from 0.162 eV to 0.318 eV, quite close to that for the adsorption of oxygen molecules on pristine graphene (∼0.15 eV)^51^ but significantly higher than that for the adsorption of hydrogen on graphene/h-BN (0.062 eV).^52^Table 3 shows that the adsorption energies of the gases on pristine graphene or graphene/h-BN increase in the following order: vdW-DF2 < revPBE-vdW < optPBE-vdW. Because the revPBE-vdW functional can provide the distance between gas molecules and graphene that is closest to the experimental value, we only present the adsorption energies of the gases on pristine graphene and graphene/h-BN using the revPBE-vdW functional in Fig. 5 and 6 in which the absolute values of the adsorption energies are arranged in an increasing trend as follows: CO < NO < CO_2_ < NO_2_. It is noteworthy that the adsorption energies increase when introducing the h-BN substrate. This implies that the combination of graphene with h-BN can enhance the slight sensitivity of graphene to toxic gases, indicating the influence of the h-BN substrate on the adsorption properties of graphene to the four toxic gases. The greatest increase in adsorption energy magnitude, due to the introduction of the two-dimensional h-BN, was 13.78% for NO. In the case of graphene/h-BN, the adsorption energy magnitude was smallest for the CO molecule at 0.171 eV and largest for the NO_2_ molecule at 0.314 eV. These values are moderate, again confirming the physisorption of graphene/h-BN to these gases, like pristine graphene. Clearly, h-BN can be considered a good substrate for increasing the mechanical stability of gas sensors, in which adsorption occurs on the surface of graphene.

We calculated the WFs of gas + graphene and gas + graphene/h-BN systems. As listed in Table 3, there is a small difference between the WFs calculated for gas + graphene and gas + graphene/h-BN in almost all cases. However, in the case of NO_2_ gases, we observed a noticeable difference between these two substrates, indicating the considerable influence of the h-BN substrate on the WFs of graphene-gas systems.

The change in the electrical signal of graphene or graphene/h-BN, caused by the charge transfer inside the adsorption systems, can be exploited to detect the presence of toxic gases. Table 3 presents the charge transfer values of gas + graphene and gas + graphene/h-BN. It shows that four toxic gases function as electron acceptors, implying the reduction of the conductivity of the graphene/h-BN upon gas adsorption. The charge transfer amount in increasing order is as follows: CO < CO_2_ < NO < NO_2_, in quantitative agreement with the fact that the shortest distances between the adsorbed gas molecule and the graphene are in the inverse order. Furthermore, the charge transfers from the adsorbents to NO and NO_2_ molecules are much greater than those to CO and CO_2_ molecules. This predicts a considerable reduction in the electrical signal of graphene and graphene/h-BN caused by the adsorption of NO and NO_2_. The calculated results of charge transfer exhibit the high selectivity of graphene for NO and NO_2_ in the cases without and with a monolayer h-BN. Table 3 indicates that upon introducing h-BN below the graphene, CO, CO_2_, NO, and NO_2_ gases received 0.007 *e*, 0.011 *e*, 0.049 *e*, and 0.196 *e*, respectively. Compared to the adsorption of these gases on pristine graphene, the electron transfer amount from the graphene/h-BN to adsorbed gas molecules decreased, excluding the increase in the electron transfer amount of 1% from the adsorbent to the NO_2_. The charge transfer decreased by 36.4% for the adsorption of CO_2_ and by 14.3% for the adsorption of CO. Possibly, the increase in the WFs due to the formation of graphene/h-BN heterostructures, as mentioned above, leads to the impairment of electron transfer. The decrease in the electron transfer amount implies that graphene/h-BN heterostructures are more inert to CO_*x*_ (*x* = 1, 2) gases as compared to pristine graphene. For NO_2_ gas, because there is an increase in the electron transfer amount from the adsorbent to the NO_2_, it can be predicted that the graphene/h-BN heterostructure is more selective toward NO_2_ than pristine graphene. Although the electron transfer amount from graphene/h-BN to NO decreased by 13.4% as compared to that from pristine graphene to NO molecule, the charge transfer amount is quite significant. Therefore, graphene/h-BN retained a good selectivity for NO as compared to pristine graphene. Graphene/h-BN heterostructures can be exploited as good gas sensing devices to distinguish NO_*x*_ and CO_*x*_ as indicated in a previous report.^53^

The recovery time *τ* is an important parameter for evaluating the performance of gas sensors. It is the time required for gas molecules to dislodge from the material surface, calculated as follows:^8^6
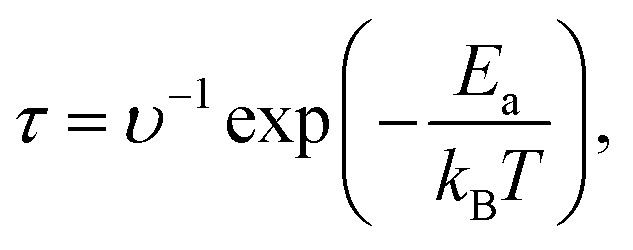
in which *υ*, *k*_B_, and *T* are the frequency, Boltzmann constant, and temperature, respectively. The calculated values of the recovery times at 300 K for the interaction of toxic gases on graphene or graphene/h-BN are presented in Table 3. These values are quite short and depend on the used functionals. The recovery times of the four gases on graphene increased slightly upon introducing the h-BN substrate. On graphene and graphene/h-BN, the recovery time of NO_2_ was the longest, about a hundred nanoseconds, while the recovery times of the three last gases were on the nanosecond scale. The short recovery time implies the almost instantaneous desorption of the four toxic gases from graphene or graphene/h-BN, which is beneficial for the application of graphene/h-BN as a good material for gas sensor devices.

**Fig. 7:**
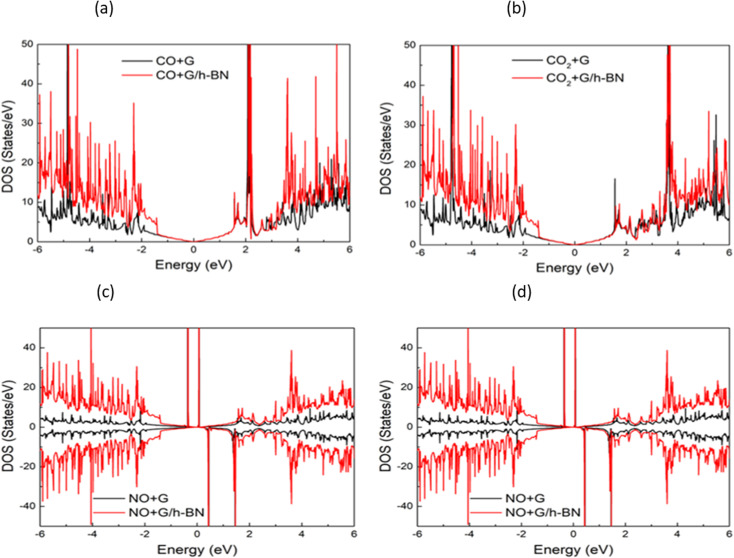
Comparison of total density of states of graphene and graphene/h-BN with different toxic gas adsorption: NO (a), NO_2_ (b), CO (c), and CO_2_ (d).

In the context of the adsorption of toxic gases on graphene and the graphene/h-BN bilayer, comparing the DOS can provide insights into the changes in the electronic structure of the system upon adsorption. Fig. 7 displays the spin polarization of graphene and graphene/h-BN upon the adsorption of NO and NO_2_, while this effect does not occur for the adsorption of CO and CO_2_. In other words, the introduction of monolayer h-BN does not cause spin polarization for the adsorption of CO and CO_2_ but it modifies the spin polarization upon the adsorption of NO and NO_2_ with the appearance of novel polarized states in high densities around the Fermi level. Because any changes in the electronic band structure may affect the adsorption behavior, the highest increase in the adsorption energy of 13.78% for NO, due to the introduction of two-dimensional h-BN, could be affected by the strong change in spin polarization.

To further explain the physical origin of the interaction of the adsorption systems and evaluate the influence of h-BN on the adsorption behavior of graphene, we performed periodic energy decomposition analyses (PEDA)^54,55^ implemented in the Amsterdam Modelling Suite^36^ to calculate the intrinsic bond energies Δ*E*_int_ and their partial energies.

7 Δ*E*_int_ = Δ*E*_eslast_ + Δ*E*_Pauli_ + Δ*E*_orb_,

where Δ*E*_elstat_ is electrostatic energy, Δ*E*_Pauli_ stands for the Pauli repulsion energy, and Δ*E*_orb_ is the orbital relaxation energy of graphene/h-BN + gas and graphene + gas. These are the measurements of the total energies of the adsorbed systems that decomposed into the corresponding energies to provide insights into the influence of the h-BN sheets on the bonding interactions between the adsorbed gas molecule and the graphene. The introduction of h-BN sheets modifies the intrinsic bond energies and their partial parts, *i.e.*, modifying the interaction between graphene and the toxic gases. Therefore, the sensitivity of graphene/h-BN to toxic gases differs from that of pristine graphene. However, the presence of the h-BN layer does not change the response trend of graphene to the considered toxic gases. With and without h-BN sheets, the Pauli repulsion energies increased in the same order: CO < CO_2_ < NO_2_ < NO. This result is consistent with the calculated shortest distances, classed as NO < NO_2_ < CO_2_ < CO, shown in Table 3. We paid great attention to the orbital energies, which are the most dominant factor in the adsorption system as shown in Table 4, which illustrates that the interactions between the absorbent and adsorbate of the two adsorption systems are driven by the orbital relaxation effect. The orbital energies for the adsorption of NO and NO_2_ are much larger than those for the adsorption of CO and CO_2_. The strong orbital relaxation is related to the spin polarization within the adsorption systems of NO and NO_2_. The orbital energy for the adsorption of NO is ∼3.1 larger than that of NO_2_. This is because the nitrogen atom of the NO molecule points down to the graphene at a distance of 3.11 Å and hybridizes with carbon atoms of the graphene as compared to the nitrogen atom of the NO_2_ molecule at a farther distance. The introduction of monolayer h-BN modifies the orbital interaction for the NO adsorption system; consequently, remarkably enhancing the spin polarization as indicated in Fig. 7(c). As a result, the adsorption energy of graphene/h-BN + NO showed the greatest increase.

**Table 4:** The periodic energy decomposition analyses (PEDA) of graphene/h-BN and pristine graphene upon the adsorption of toxic gases. All units are in J mol^−1^

Substrate	PEDA types	CO	CO_2_	NO	NO_2_
Graphene/h-BN	Δ*E*_int_	−39	−48	−479	−156
	Δ*E*_Pauli_	35	53	151	108
	Δ*E*_elstat_	−32	−56	−75	−84
	Δ*E*_orb_	−42	−45	−556	−180
Graphene	Δ*E*_int_	−47	−66	−545	−210
	Δ*E*_Pauli_	34	53	244	90
	Δ*E*_elstat_	−43	−76	−144	−93
	Δ*E*_orb_	−37	−43	−645	−207

#### 3.3.3. The transport properties of graphene/h-BN heterostructures upon the adsorption of toxic gases

**Fig. 8:**
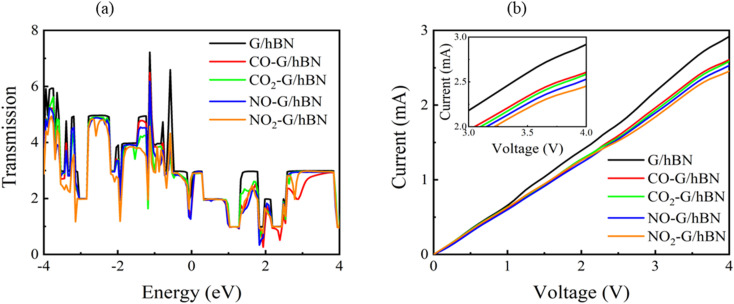
Transmission spectra at zero bias voltage (a) and current–voltage profile (b) of graphene/h-BN heterostructures upon the adsorption of toxic gases.

To evaluate the transport properties of the graphene/h-BN heterostructures upon the adsorption of toxic gas molecules, we placed the most stable gas + graphene/h-BN configurations as the scattering region between two drain electrodes of graphene/h-BN in which these components are in the same plane as displayed in Table 5. We also calculated the electron transport properties of these corresponding pristine bilayers for further comparison. The transmission spectra at zero bias voltage are presented in Fig. 8(a). The black, red, green, blue, and orange curves indicate the transmission of the graphene/h-BN, CO + graphene/h-BN, CO_2_ + graphene/h-BN, NO + graphene/h-BN, and NO_2_ + graphene/h-BN, respectively. As shown in Fig. 8, in general, the transmission spectra of four gas + graphene/h-BN systems are in the same form in the energy range from −4 eV to 4 eV. Upon the adsorption of gas molecules, due to the charge transfer from these molecules to the substrates, these heterostructures hindered the free electron mobility through the scattering regions resulting in a reduction of the electrical conductivity, in quantitative agreement with a recent report.^53^ Consequently, we observed a remarkable decrease in current passing through these channels upon the adsorption of toxic gases under the bias voltage beyond 3 V. Essentially, the more charge is transferred from the gas molecules to the substrates, the faster is the reduction of electrical current beyond 3 V applied voltage. The electrical behavior of the *I*–*V* profile agrees well with the charge transfer presented in Table 3. These results demonstrate the sensitivity and selectivity of toxic gas molecules adsorbed on the bilayer systems.

**Table 5:** Electron transport schematic models and shapes of frontier orbitals of graphene/h-BN without and with toxic gas molecules on the surface

Sample	Scattering model, HOMO and LUMO shapes
G/hBN	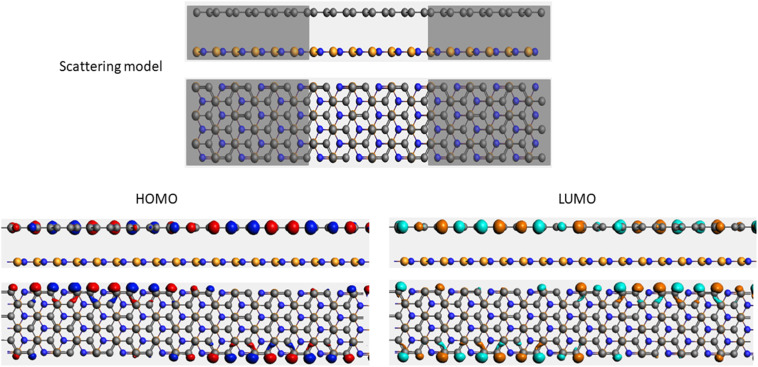
CO + G/hBN	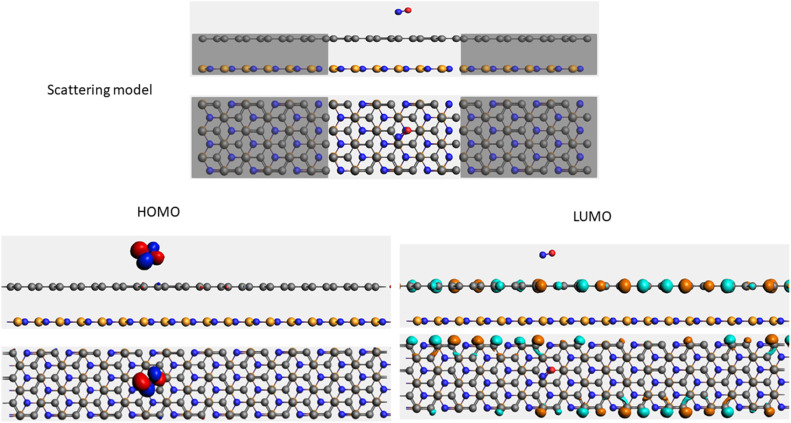
CO_2_+ G/hBN	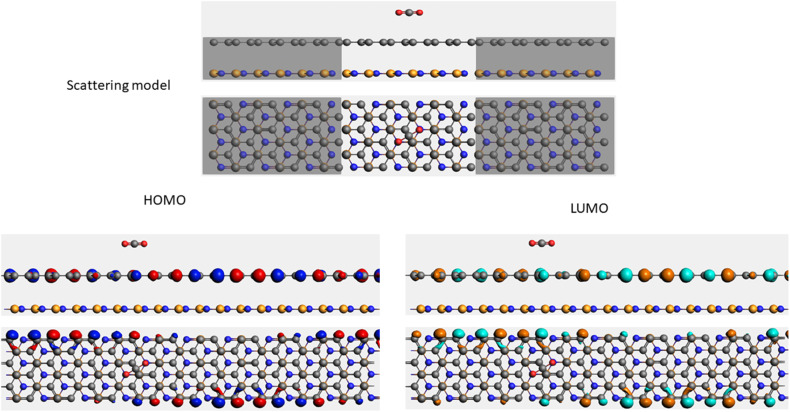
NO + G/hBN	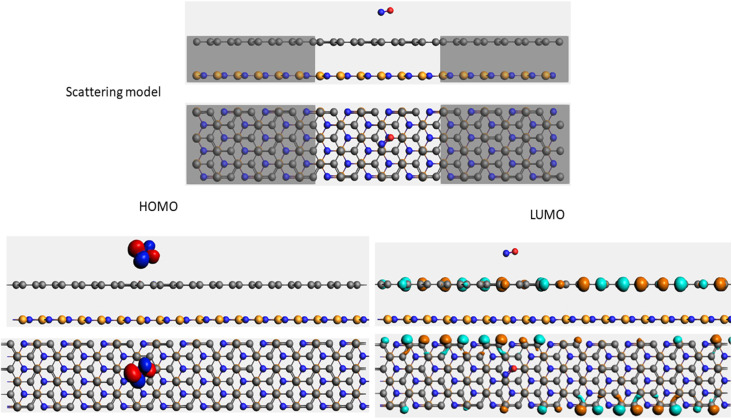
NO_2_ + G/hBN	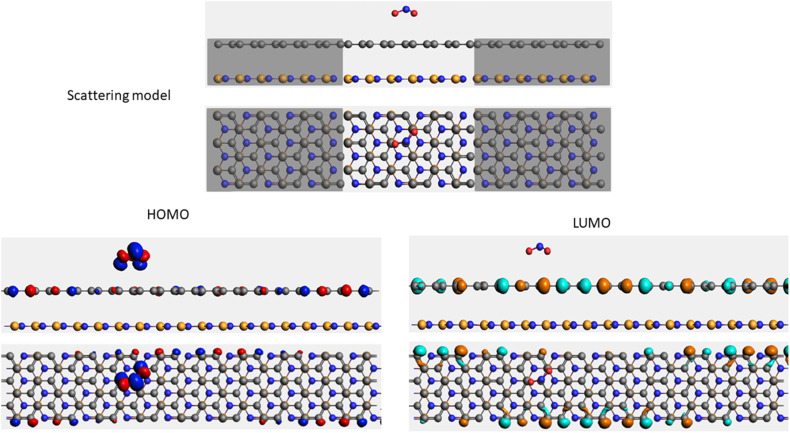

It is well-known that the electronic transport properties of materials are governed by the electrons of the frontier orbitals. To gain further insight into the effects of toxic gas adsorption on the electronic properties of graphene/h-BN heterostructure GQDs, we analyzed the modification of the HOMO and LUMO shapes of the most stable graphene/h-BN due to the interaction with the gas molecules. The LUMO (HUMO) shapes are displayed in Table 5, in which the charge accumulation regions are in red (orange), whereas the charge depletion areas are in blue (green). As shown in Table 5, the LUMO schema of the scattering region changed inconsiderably, whereas the HOMO of graphene/h-BN heterostructures exhibited a considerable modification. The interaction between graphene/h-BN heterostructures and gas molecules leads to the redistribution of electrons of the HOMO on the surfaces of the scattering regions. The electrons of the HOMO of the graphene/h-BN upon adsorption of CO, NO, and NO_2_ are located around the adsorbed molecules instead of being distributed at the edge of the scattering region for the graphene/h-BN without gas molecules on the surface. For the adsorption of CO_2_, the electron density of the HOMO was observed to decrease with respect to that of the of the graphene/h-BN. This indicates a transfer of electrons from the HOMO state of graphene to the adsorbed molecules. The redistribution of the electrons of the HOMO state caused by the gas adsorption led to a decrease in the current signals as illustrated in Fig. 8(b).

## 4. Conclusions

We have presented a systematic study on the effects of h-BN substrate on the gas adsorption performance of graphene *via* first-principles calculations using vdW functionals. We found that embedding graphene on the top of a two-dimensional monolayer h-BN boosts the mechanical stability with a small mismatch and tunes the electronic properties of graphene, including a gap opening of 38 meV and an increase in the work function. The changes in the electronic properties positively impact the adsorption performances of graphene toward CO, CO_2_, NO, and NO_2_. When the orbital relaxation energy is the dominant factor, the introduction of the h-BN substrate changes the orbital relaxation, enhancing the sensitivity of graphene to NO_2_, such as increasing the adsorption energies and the reduction of the shortest distances between the graphene/h-BN and the adsorbed gas molecules. The graphene/h-BN heterostructure exhibited a greater selectivity for NO_2_ gas and inertness toward CO_*x*_ gases. Although there were several changes in the gas adsorption properties with respect to pristine graphene, the graphene/h-BN heterostructure still exhibited physisorption to toxic gases similar to pristine graphene. The conductivity of graphene/h-BN heterostructures is predicted to remarkably decrease when adsorbing toxic gases because the adsorbed molecules work as charge acceptors. The decrease in the conductivity strongly depends on the type of adsorbed gas, indicating the selectivity of the graphene/h-BN heterostructures. The findings show the potential for the application of graphene and h-BN heterostructures as room-temperature gas sensors with high performance.

## Author contributions

All authors have approved the final version of the manuscript.

## Conflicts of interest

The authors declare no competing financial interest.

## Supplementary Material
